# Supplementary material for: Immobilization of Dextranase on Nano-Hydroxyapatite as a Recyclable Catalyst
Source: Materials (Basel). 2020 Dec 30;14(1):130. doi: 10.3390/ma14010130 (PMC7796272; doi:10.3390/ma14010130)
Supplement: Supplementary file 1 [file materials-14-00130-s001.pdf]

Supporting information

Immobilization of dextranase on nano-hydroxyapatite as a recyclable catalyst

Yanshuai Ding[a,b], Hao Zhang[a,b],Xuelian Wang[a,b],Hangtian Zu[a,b],Cang Wang[a,b],Dongxue Dong[a,b], Mingsheng Lyu [a,b,c,], Shujun Wang[a,b,c,*]

[a]Jiangsu Key Laboratory of Marine Bioresources and Environment /Jiangsu Key Laboratory of Marine Biotechnology, Jiangsu Ocean University, Lianyungang, 222005, PR China

[b]Co-Innovation Center of Jiangsu Marine Bio-industry Technology, Jiangsu Ocean University, Lianyungang, 222005, PR China

[c]Collaborative Innovation Center of Modern Biological Manufacturing, Anhui University, Hefei 230039, China

Methods

Particle size

The nano-hydroxyapatite (HA) was dispersed in water and sonicated. Then, the size was detected by dynamic light scattering (DLS) (HORIBA Scientific).

Zeta potential of HA

Ten mg of HA was dispersed in 50 mL of pure water, and the solution was sonicated for 10 min. The solution mixed with 100 mM buffer one by one to adjust the pH (measured in pH range of 4-9), and the Zeta potential of HA was measured (Zetasizer Nano ZS90, Malvern UK). The data represents the average of three runs for each sample.

Results

The average size was 32 nm +/- 6.7 nm (Figure S1).

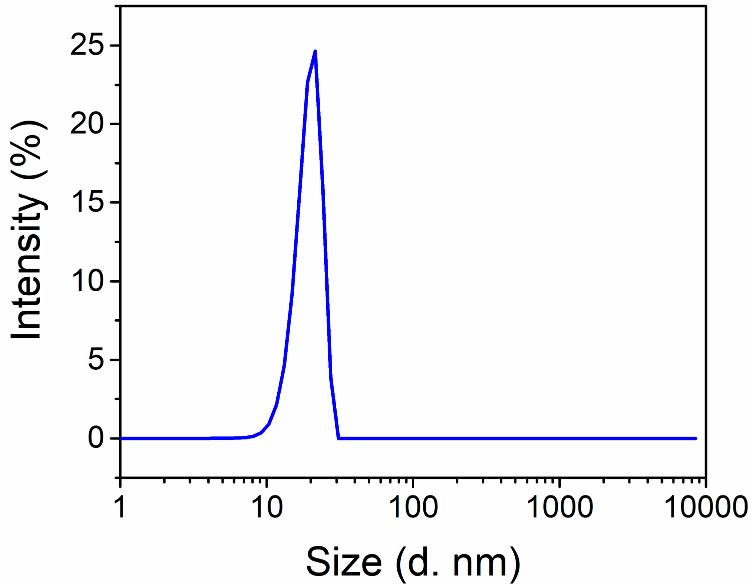

Figure S1. The size of HA NPs detected by dynamic light scattering (DLS)

The HA NPs showed negative potential in different pH condition (Figure S2).

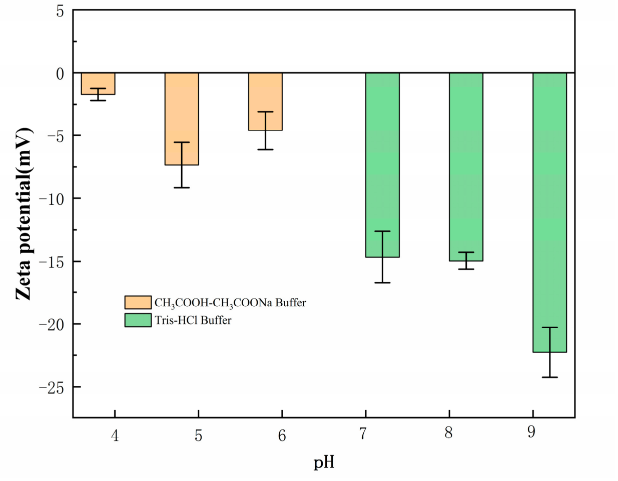

Figure S2 Zeta potentials of HA

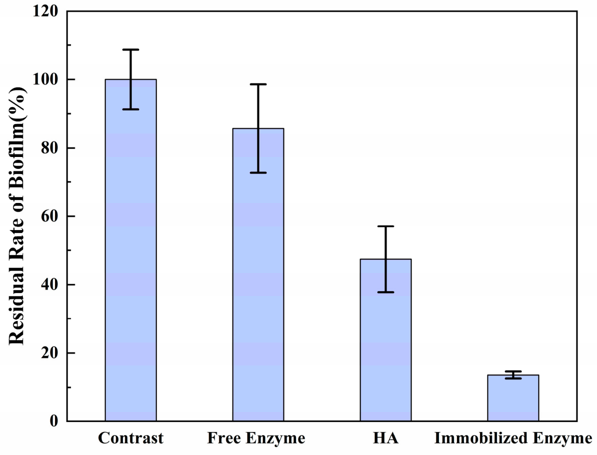

Figure S3. Residual rate of biofilm after different additives on dental plaque biofilm (pure water served as the control group)
